# FGF signalling through RAS/MAPK and PI3K pathways regulates cell movement and gene expression in the chicken primitive streak without affecting E-cadherin expression

**DOI:** 10.1186/1471-213X-11-20

**Published:** 2011-03-21

**Authors:** Katharine M Hardy, Tatiana A Yatskievych, JH Konieczka, Alexander S Bobbs, Parker B Antin

**Affiliations:** 1 Department of Cell Biology and Anatomy, University of Arizona, Medical Research Building, 1656 E. Mabel Street, Tucson, AZ 85724, USA; 2 Department of Cellular and Molecular Biology, University of Arizona, 1007 E. Lowell Street Tucson, AZ 85721, USA; 3 Program in Cancer Biology and Epigenomics, Children's Memorial Research Center, Northwestern University Feinberg School of Medicine, 2300 Children's Plaza, Box 222, Chicago, IL 60614, USA; 4 Department of Molecular and Cellular Biology, Harvard University, Cambridge, MA 02138, USA

## Abstract

**Background:**

FGF signalling regulates numerous aspects of early embryo development. During gastrulation in amniotes, epiblast cells undergo an epithelial to mesenchymal transition (EMT) in the primitive streak to form the mesoderm and endoderm. In mice lacking FGFR1, epiblast cells in the primitive streak fail to downregulate E-cadherin and undergo EMT, and cell migration is inhibited. This study investigated how FGF signalling regulates cell movement and gene expression in the primitive streak of chicken embryos.

**Results:**

We find that pharmacological inhibition of FGFR activity blocks migration of cells through the primitive streak of chicken embryos without apparent alterations in the level or intracellular localization of E-cadherin. E-cadherin protein is localized to the periphery of epiblast, primitive streak and some mesodermal cells. FGFR inhibition leads to downregulation of a large number of regulatory genes in the preingression epiblast adjacent to the primitive streak, the primitive streak and the newly formed mesoderm. This includes members of the FGF, NOTCH, EPH, PDGF, and canonical and non-canonical WNT pathways, negative modulators of these pathways, and a large number of transcriptional regulatory genes. *SNAI2 *expression in the primitive streak and mesoderm is not altered by FGFR inhibition, but is downregulated only in the preingression epiblast region with no significant effect on E-cadherin. Furthermore, over expression of SNAIL has no discernable effect on E-cadherin protein levels or localization in epiblast, primitive streak or mesodermal cells. FGFR activity modulates distinct downstream pathways including RAS/MAPK and PI3K/AKT. Pharmacological inhibition of MEK or AKT indicate that these downstream effectors control discrete and overlapping groups of genes during gastrulation. FGFR activity regulates components of several pathways known to be required for cell migration through the streak or in the mesoderm, including RHOA, the non-canonical WNT pathway, PDGF signalling and the cell adhesion protein N-cadherin.

**Conclusions:**

In chicken embryos, FGF signalling regulates cell movement through the primitive streak by mechanisms that appear to be independent of changes in E-cadherin expression or protein localization. The positive and negative effects on large groups of genes by pharmacological inhibition of FGF signalling, including major signalling pathways and transcription factor families, indicates that the FGF pathway is a focal point of regulation during gastrulation in chicken.

## Background

Vertebrate gastrulation is a highly coordinated process that leads to formation of the three primary germ layers (ectoderm, mesoderm and endoderm) and sets up the body plan for subsequent organ development. The morphogenetic aspects of gastrulation vary considerably across different groups of organisms. In general, cells in an outer embryo layer move inward to form the mesoderm and the endoderm, while simultaneously large-scale cell movements and changes in cell shape transform overall embryo structure [1,2].

A defining feature of gastrulation in amniotes (reptiles, birds and mammals) is that mesoderm cells arise from the epithelial epiblast through an EMT in the primitive streak [3,4]. This contrasts with mesoderm development in lower vertebrates such as frogs and fish in which presumptive mesodermal cells involute and migrate as a generally contiguous sheet. In chicken, the primitive streak arises following dramatic polonaise cell movements within the epiblast, leading to cell intercalation in the preingression epiblast region [5-7].

Primitive streak formation and the emergence of endoderm and mesoderm is closely integrated with changes in cell fate. Both processes are regulated by several growth factor signalling pathways, including the canonical and non-canonical WNT, PDGF, BMP, NODAL, and FGF pathways [5,6,8-12]. In situ hybridization (ISH) analyses have shown that members of multiple signalling pathways are expressed in the primitive streak regions of gastrula stage chicken embryos [13-20]. Some of these pathways, as well as other mechanisms, regulate cell migration in the primitive streak [16,18,21-23].

FGF signalling is an important mediator of mesoderm induction and gastrulation movements. FGFs can induce mesoderm in frog animal caps and avian epiblast [24-26]. Mouse embryos lacking *FgfR1 *initially form a streak, but cells fail to undergo EMT due to the absence of *Snai1 *expression and failure to downregulate E-cadherin [27]. The downregulation of E-cadherin via transcriptional repression by Snail proteins is considered a prerequisite for EMT in many contexts [28,29], including during mouse gastrulation [27].

In chicken embryos, FGFR1 signalling is necessary for the primitive streak to form [6,30,31]. Following emergence of mesoderm cells from the primitive streak, FGFs appear to act as chemotactic factors that influence mesoderm migration. Mesoderm cells will migrate towards a source of FGF4 but away from FGF8 [21]. In mouse embryos lacking *Fgf8*, emerging mesoderm cells gastrulate but fail to migrate away from the primitive streak [32]. Together, these findings indicate that FGF signalling plays a primary role in regulating primitive streak formation, mesoderm induction, and mesoderm migration.

In this study, we investigate how FGF signalling and its downstream effectors regulate cell movement and gene expression in and around the primitive streak of chicken embryos after the onset of gastrulation. In contrast to results of genetic ablation studies in mice [27], pharmacological inhibition of FGFR activity blocks migration of cells through the primitive streak of chicken embryos by mechanisms that appear to be independent of E-cadherin localization or expression levels. E-cadherin protein levels are high throughout the epiblast, in cells undergoing EMT, and in the newly formed mesoderm, and are unaffected by over expression of SNAIL. FGFR inhibition leads to downregulation of a large number of regulatory and effector genes through both the RAS/MAPK and PI3K/AKT pathways.

## Results

### Regulatory gene expression in gastrula stage chicken embryos

**Figure 1:**
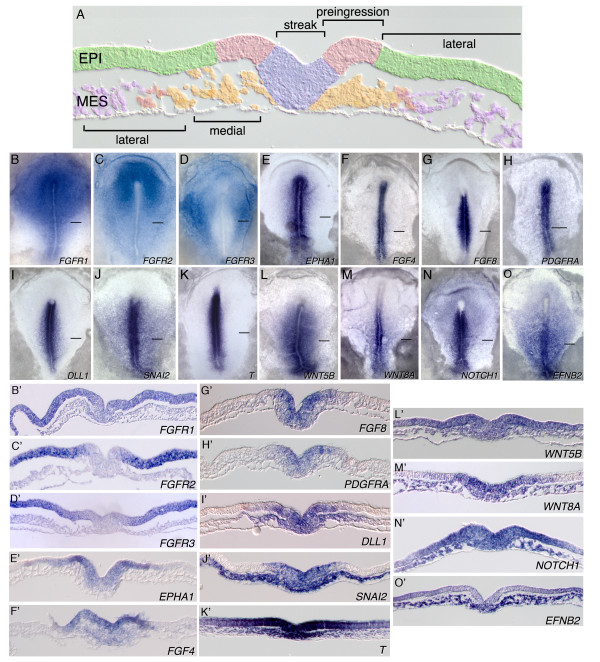
**Domains of gene expression in the gastrula stage chicken embryo**. A: Transverse section through the mid-streak region of a stage 4 embryo depicting domains represented by the expression patterns in B-O, B'-O' (green, lateral epiblast; pink, preingression epiblast; lavender, primitive streak; orange, medial mesoderm; purple, lateral mesoderm). B-O: Whole mount ISH localization of mRNAs coding for signalling molecules, receptors and growth factors in stage 4 embryos. B'-O': Transverse sections at the indicated levels through corresponding embryos in B-O. Abbreviations: EPI, epiblast; MES, mesoderm.

To obtain an overview of regulatory gene expression patterns in the primitive streak, stage 4 embryos were assayed by ISH for expression of a candidate group of transcription factors, growth factors, and receptors. Analysis of whole embryos and transverse embryo sections identified several patterns that can be described by combinatorial expression in one or more of the following morphological domains: lateral epiblast, preingression epiblast, primitive streak, medial mesoderm, and lateral mesoderm (Figure 1A). For example, *FGFR1 *is expressed in the lateral epiblast, the preingression epiblast, and the primitive streak, but at greatly reduced levels in the newly formed mesoderm (Figure 1B, B'). *FGFR2 *and *FGFR3 *transcripts are detected in the lateral epiblast but at much lower levels in the preingression epiblast and primitive streak (Figure 1C-D, C'-D'). Genes such as *EPHA1, FGF4, FGF8*, *PDGFRA*, and *DLL1 *are expressed in the preingression epiblast and primitive streak, and then downregulated in the mesoderm (Figure 1E-I, E'-I'; for this study, the preingression epiblast is defined as the domain of epiblast adjacent to the primitive streak that expresses these genes). *SNAI2 *shows a similar expression pattern except that transcripts persist to more lateral regions of the mesoderm (Figure 1J, J'). *T*, *WNT5B*, *WNT8A*, and *NOTCH1 *are expressed in the preingression and more lateral epiblast, the primitive streak, and the mesoderm extending to the lateral regions (Figure 1K-N, K'-N'). Finally, genes such as *EFNB2 *are expressed in the primitive streak and broadly in the mesoderm (Figure 1O, O').

### FGF signalling is required for cell migration through the primitive streak

Several observations suggest that FGF signalling is active in the preingression epiblast and primitive streak. First, *FGF3, FGF4, FGF8, FGF13*, and *FGF19 *are expressed in these domains (Figure 1F, G; [33]). Second, *FGFR1 *transcripts are detected in the lateral and preingression epiblast and in the primitive streak, but at low or undetectable levels in the emerging mesoderm (Figure 1B, B'; [17,20]). *FGFR2 *and *FGFR3 *are expressed at high levels in the lateral epiblast but at much lower or undetectable levels in the preingression epiblast, primitive streak, and mesoderm (Figure 1C-D, C'-D'). *FGFR4 *transcripts are detected only in extraembryonic regions [17,20]. Third, activated ERK (dpERK), an indicator of FGF signalling, is detected in the preingression epiblast and the primitive streak, with much lower or undetectable levels in emerging mesoderm [17]. While RNA localization may not reflect protein expression, these results nevertheless suggest that the FGFR1 receptor is present and active in the primitive streak.

To determine if FGFR activity is required for cell migration through the primitive streak, stage 3d-4 embryos were pretreated for two hours with the FGFR inhibitor SU5402 [34,35] or with DMSO as a control, and then electroporated with a *GFP *expression plasmid [36]. Extensive control experiments have shown that this electroporation protocol specifically targets cells in the epiblast [16], and so assaying for GFP-positive cells in the mesoderm following a period of development reflects the ability of cells to move from the epiblast through the primitive streak. The concentration of SU5402 used (100 μM) was determined by preliminary titration studies to assess the minimum concentration that would abolish detectable expression of *T *(Brachyury) by ISH and phospho-ERK by western blot.

**Figure 2:**
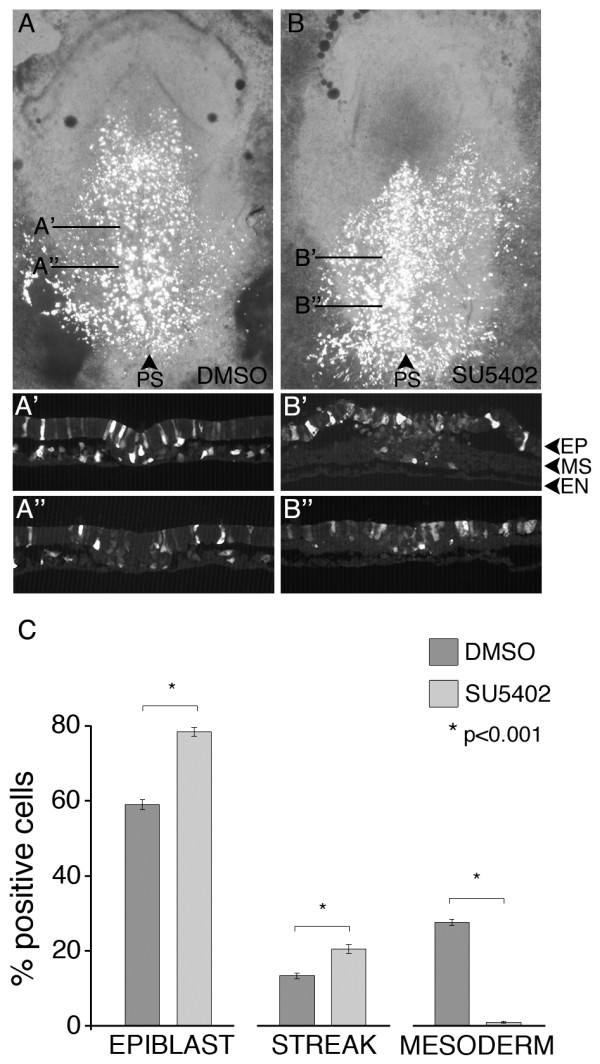
**Inhibition of FGFR activity blocks cell migration from the epiblast through the primitive streak to the mesoderm**. A-B: Combined brightfield and fluorescence images of control (A) or SU5402 treated (B) embryos. Embryos were treated for two hours with DMSO carrier or SU5402, and then electroporated with a GFP expression construct. A'-A": indicates transverse sections through the control embryo in A; B'-B": represents transverse sections through the embryo shown in B. C: Quantification of GFP-expressing cell location at 5 hours after electroporation. EPIBLAST includes preingression epiblast and lateral epiblast extending to the area pellucida-opaca border; STREAK includes the primitive streak; MESODERM includes the medial and lateral mesoderm regions to the area pellucida-opaca border (see Figure 1A for depiction of domains). Migration of cells to the mesoderm is essentially abolished in SU5402 treated embryos. Abbreviations: PS, primitive streak; EP, epiblast; MS, mesoderm; EN, endoderm.

Analysis of GFP-positive cells in control embryos five hours following electroporation showed typical migration patterns of cells through the primitive streak (Figure 2A-A"). GFP-positive cells were distributed in the lateral and preingression epiblast, primitive streak, and mesoderm layers. In contrast, GFP-positive cells in SU5402-treated embryos were present in the epiblast and primitive streak regions but were rarely observed in the mesoderm layer (Figure 2B-B"). Cell counts indicated that significantly more positive cells were retained in the epiblast (lateral plus preingression regions; 78.5% versus 59.0% respectively; p < 0.001) and primitive streak (20.6% versus 13.4%; p < 0.001) in SU5402 versus DMSO treated embryos, while contribution to the mesoderm was virtually abolished by SU5402 (1.0% versus 27.6%; p < 0.001; Figure 2C). This data indicates that FGFR activity is required for cells to transition from the epiblast through the primitive streak to populate the mesoderm.

### FGF receptor activity is necessary for regulatory gene expression in the primitive streak

**Figure 3:**
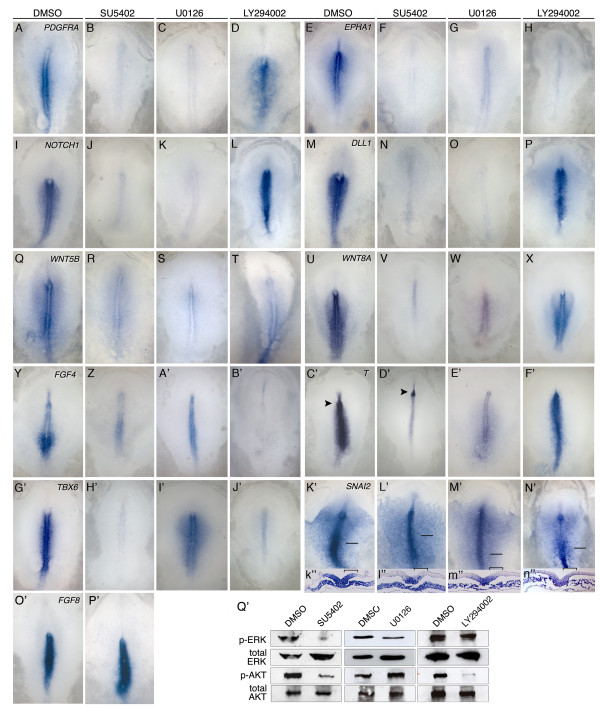
**Effects of SU5402, the MEK inhibitor U0126, and the PI3K inhibitor LY294002 on gene expression in the primitive streak region**. A-P': Whole mount images showing mRNA expression in control (DMSO), SU5402, U0126 and LY294002 treated embryos. Arrows in C'-D' point to Hensen's node. Brackets in k"-l" indicate the preingression epiblast. Q': Western blot analysis comparing total versus phosphorylated ERK1/2 (p-ERK) and AKT (p-AKT) in the preingression epiblast and primitive streak of DMSO versus SU5402, U0126 and LY294002 treated embryos.

Components of numerous pathways require FGFR activity for expression. Expression of the receptors *PDGFRA*, *EPHA1*, and *NOTCH1 *(Figure 3A-B, E-F, I-J), and the ligands *DLL1, WNT5B, WNT8A*, and *FGF4 *(Figure 3M-N, Q-R, U-V, Y-Z), were significantly reduced in embryos exposed to SU5402. Surprisingly, *FGF8 *mRNA levels were unchanged or slightly elevated by SU5402 treatment (Figure 3O'-P'). The T-box transcription factor *T *was downregulated in the primitive streak but not in Hensen's node or the notochord, (Figure 3C'-D'), while expression of *TBX6 *was globally downregulated by SU5402 treatment (Figure 3G'-H'). Whereas *SNAI2 *was expressed in the preingression epiblast, primitive streak, and mesoderm in control embryos (Figure 3K'-k"), SU5402 treatment inhibited *SNAI2 *expression only in the preingression epiblast (Figure 3L'-l").

**Figure 4:**
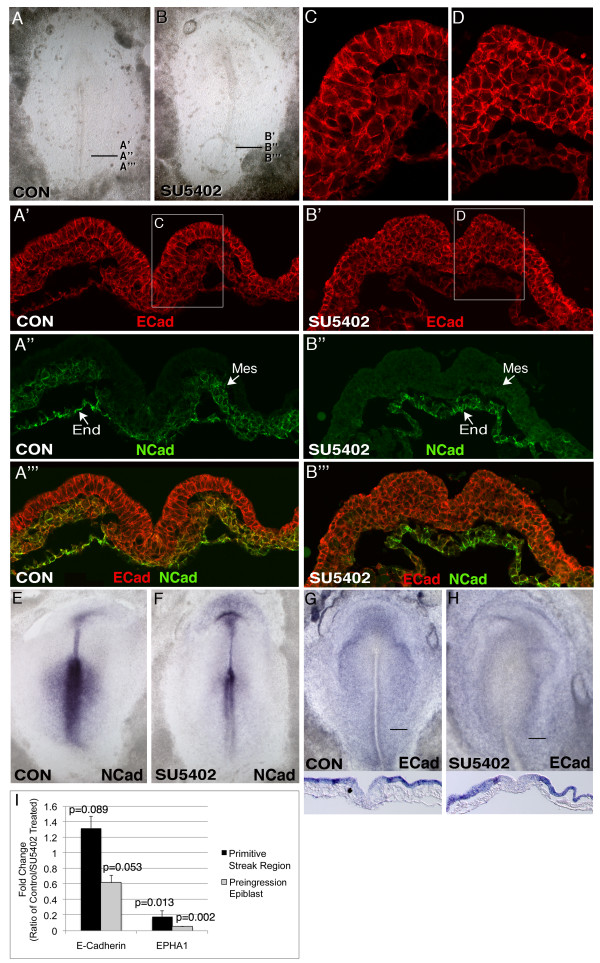
**E- and N-cadherin expression in control and SU5402 treated embryos**. A-B: Brightfield images of control (A) and SU5402 treated (B) embryos that were processed for immunofluorescence analysis of E-cadherin (ECad) and N-cadherin (NCad). A'-A'": transverse section showing the same microscopic field at the indicate streak level of the control embryo in A, visualizing E-cadherin protein (A'), N-cadherin protein (A"), and E-cadherin plus N-cadherin (A'"). B'-B'": transverse section showing the same microscopic field at the indicate streak level of the SU5402 treated embryo in B, visualizing E-cadherin protein (B'), N-cadherin protein (B"), and E-cadherin plus N-cadherin (B'"). C-D: higher magnification views of the boxed areas in A' and B'. E-H: Whole mount ISH visualization of N-cadherin (E, F) and E-cadherin (G, H) mRNAs in control and SU5402 treated embryos. I: Realtime RT-PCR analysis showing relative E-cadherin and EPHA1 mRNA levels in control versus SU5402 treated primitive streak region (preingression epiblast, primitive streak, medial mesoderm) and isolated preingression epiblast. Error bars indicate standard deviation. Abbreviations: Mes, mesoderm; End, endoderm.

The SNAIL transcription factors are widely regarded as key regulators of EMT through their ability to downregulate E-cadherin in epithelial cells [29]. In mice, embryos lacking *FgfR1 *fail to express *Snai1 *in the primitive streak, leading to the persistence of E-cadherin expression and failure of cells to exit the epiblast and migrate through the primitive streak [27]. Since SU5402 abrogates *SNAI2 *expression only in the preingression epiblast (Figure 3K'-l"), we investigated the effects of SU5402 treatment on E-cadherin mRNA and protein levels and localization. In control embryos, E-cadherin protein was localized primarily to the periphery of all cells in the epiblast, primitive streak, and medial mesoderm (Figure 4A-A', A'", and 4C; Additional file 1, Figure S1A-G). E-cadherin labelling in ventral streak cells remained high, while mesodermal cells near the streak showed slightly reduced E-cadherin staining intensity that remained primarily localized to the cell periphery (Figure 4C). In posterior regions of control embryos, E-cadherin labelling was observed throughout the mesoderm layer, while in more anterior regions, E-cadherin levels were reduced in the lateral mesoderm (Additional file 1, Figure S1A-G). Surprisingly, in cells of SU5402 treated embryos, neither the levels nor the localization of E-cadherin protein appeared different from controls (compare Figure 4A' and 4C with Figure 4B' and 4D; Additional file 1, Figure S1).

Although E-cadherin labelling patterns were indistinguishable between control and SU5402 treated embryos (compare Figure 4A' and 4C with Figure 4B' and 4D; Additional file 1, Figure S1), striking differences in epiblast cell morphology were apparent between the groups. Cells in the preingression epiblast of control embryos exhibited the typical, highly polarized epithelial morphology (Figure 4A' and 4C). However, in the posterior half of SU5402 treated embryos, cells in the preingression epiblast lacked the characteristic columnar epithelial morphology seen in normal preingression epiblast cells (compare Figure 4A' and 4C with 4B' and 4D).

In control embryos, N-cadherin protein was detected in all cells of the mesoderm and endoderm layers (Figure 4A"; Additional file 1, Figure S1A-G; [23]). In posterior regions of control embryos, N-cadherin was absent from dorsal primitive streak cells, while in more anterior regions staining was evident in some cells of the dorsal primitive streak (Additional file 1, Figure S1A-G). The relative proportions of N- and E-cadherin labelling varied between individual cells of the streak and the mesoderm layer (Figure 4A'"). In contrast to E-cadherin, N-cadherin labelling intensity was significantly reduced in the posterior mesoderm of SU5402 treated embryos compared with control embryos (contrast Figure 4B" with Figure 4A"). In agreement with this, ISH analysis showed a significant reduction of *N-cadherin *mRNA in the posterior primitive streak region of SU5402 treated embryos (Figure 4E-F). In more anterior regions, however, N-cadherin staining appeared roughly equivalent in control and SU5402 treated embryos (Additional file 1, Figure S1). N-cadherin labelling intensity was also roughly equivalent in the endoderm of control versus treated embryos (Figure 4A" versus 4B"; Additional file 1, Figure S1).

FGFR inhibition leads to *SNAI2 *downregulation in the preingression epiblast, but not in the middle to lower portions of the streak or in mesoderm cells (Figure 3K'-l"). Since SNAI2 is known to repress *E-cadherin *gene transcription, *E-cadherin *mRNA levels were also assessed by ISH and PCR analyses. By ISH, *E-cadherin *mRNA levels in control and SU5402 treated embryos appeared no different in the preingression epiblast or in other regions of the embryo (Figure 4G-H). By RT-PCR, *E-cadherin *mRNA levels were not statistically different in primitive streak or in preingression epiblast regions of control versus treated embryos (Figure 4I), though mRNA levels in the isolated preingression epiblast of SU5402 treated embryos showed a trend towards being reduced (p = 0.053). *E-cadherin *mRNA levels in the mesoderm were low but detectable in both control and treated embryos (approximately eight-fold lower than in the epiblast; data not shown). Altogether, the immunofluorescence, ISH and RT-PCR analyses fail to show an increase in E-cadherin levels following inhibition of FGFR activity, despite a reduction of *SNAI2 *mRNA in the preingression epiblast.

To further explore the relationship between SNAI2 and E-cadherin expression, the effect of SNAIL over expression on E-cadherin protein levels and localization was investigated. Three FLAG-tagged SNAIL expression vectors were utilized: wild-type chicken SNAI2 (WTcSNAI2), wild-type human SNAI1 (WThSNAI1; SNAI1 is expressed in the mammalian primitive streak), and a degradation resistant form of human SNAI1 (6SAhSNAI1) that shows an enhanced ability to downregulate E-cadherin and induce EMT [37].

**Figure 5:**
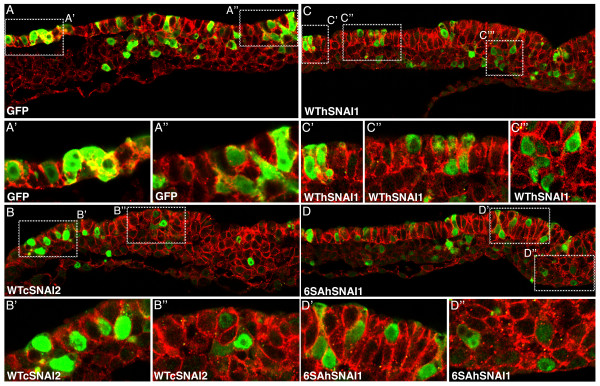
**SNAIL over expression does not alter E-cadherin protein**. Confocal microscopy images of transverse sections of embryos electroporated with a GFP expression vector (A-A"), or FLAG-tagged versions of wild type chicken SNAI2 (WTcSNAI2; B-B"), wild type human SNAI1 (WThSNAI1; C-C"), or a degradation resistant form of human SNAI1 (6SAhSNAI1; D-D"). GFP (A) or FLAG (B-D) (green) and E-cadherin (red) were visualized by indirect immunofluorescence and confocal microscopy.

The epiblast and primitive streak of stage 3d embryos was electroporated with one of the SNAIL expression vectors (or a GFP expression plasmid as a control), then incubated for 8 hours. Following fixation, embryos were assayed by dual immunofluorescence with antibodies to FLAG or GFP, and to E-cadherin. Regardless of the SNAIL construct used, over expression did not alter E-cadherin protein levels or localization (Figure 5). SNAIL-positive cells were scattered throughout the epiblast, primitive streak and mesoderm in distributions that were not different from control embryos electroporated with a GFP expression plasmid (Figure 5A-D). Regardless of the SNAIL construct electroporated, over expression did not apparently cause epiblast cells to undergo precocious EMT, since many SNAIL-positive cells were observed in the epiblast and in the primitive streak. Importantly, none of the SNAIL constructs appeared to downregulate E-cadherin protein, as SNAIL-expressing cells retained E-cadherin protein at their periphery in patterns indistinguishable from non-expressing cells or cells of embryos expressing GFP (compare Figure 5B'-D" with 5A'-A"). The ability of SNAIL expression constructs to downregulate E-cadherin was confirmed by transfection into MDCK cells (not shown). These results indicate that, within the time-course of the experiment, SNAIL over expression is insufficient to downregulate E-cadherin protein levels.

### FGF signalling during gastrulation is propagated via RAS/MAPK and PI3K/ATK pathways

FGF signalling can activate a number of downstream signalling cascades, including Ras to mitogen-activated protein kinase (RAS/MAPK) and phosphatidylinositol 3-kinase (PI3K/AKT) [38]. To determine if SU5402 affects either of these pathways, the phosphorylation states of ERK1/2 and AKT were assayed in the preingression epiblast and primitive streak regions of control versus SU5402 treated embryos. Compared with DMSO treated control embryos, SU5402 treatment markedly reduced the phosphorylation levels of ERK and AKT (Figure 3Q').

To determine the contribution of the RAS/MAPK and PI3K/AKT activity to gene expression in the primitive streak region, embryos were treated with the MEK inhibitor U0126 or the AKT inhibitor LY294002, and assayed for expression of candidate genes. Consistent with results obtained using SU5402, inhibition of MEK activity resulted in downregulation of the signalling pathway receptors *PDGFRA*, *EPHA1*, and *NOTCH1 *(Figure 3C, G, K), ligands *DLL1, WNT5B*, and *WNT8A *(Figure 3O, S, W), and the transcription factor *T *(Figure 3D'). In contrast, *FGF4 *and *TBX6 *transcript levels were unaffected by U0126 (Figure 3A', I'). As observed following SU5402 treatment, U0126 abolished *SNAI2 *expression only in the preingression epiblast while expression in the primitive streak and mesoderm was unaffected (Figure 3M', m"). Western blot analysis showed that U0126 reduced the levels of phosphorylated ERK, while levels of phosphorylated AKT were unchanged (Figure 3Q').

The expression of most FGFR and MEK-dependent genes was unaffected by AKT inhibition (Figure 3D, L, P, X, F'). However, *EPHA1*, which was abolished by both SU5402 and U0126 treatments (Figure 3F-G), and *SNAI2*, which was inhibited only in the preingression epiblast region (Figure 3L'-m"), were also downregulated by LY294002 (Figure 3H, N', n"). *FGF4 *and *TBX6 *transcript levels were unaffected by U0126 treatment (Figure 3A', I'), but were essentially abolished by treatment with LY294002 (Figure 3B', J'). Western blot analysis demonstrated that LY294002 treatment greatly reduced phosphorylated AKT levels while levels of phosphorylated ERK were unchanged (Figure 3Q'). Together, these results suggest that FGF signalling acts through both ERK and AKT to control regulatory gene expression in the preingression epiblast and primitive streak. Most FGFR dependent genes assayed required only ERK signalling for high-level expression, while expression of a few genes was dependent on signalling only through AKT. *EPHA1 *expression and *SNAI2 *expression in the preingression epiblast were dependent on both signalling pathways.

### Microarray analysis of gene expression

Considering the specific effects of FGFR, MEK, and AKT inhibition on *SNAI2 *expression in the preingression epiblast, microarray studies were performed to obtain a more comprehensive view of gene expression changes in the different epiblast expression domains illustrated in Figure 1A. First, gene expression levels were compared between lateral and preingression epiblast, excluding the primitive streak. Approximately 630 genes were upregulated in the preingression epiblast versus lateral epiblast (Table 1; Additional file 2, Table S1; adjusted p < 0.05, at least 1.5 fold change; see methods for discussion of data analysis). Genes upregulated in the preingression epiblast comprised members of several signalling pathways, including *NOTCH1, DLL1, WNT3A, WNT5B, WNT8A, EDNRB*, *EDNRB2, PDGFRA, FGF3, FGF4, FGF8, FGF18*, and *EPHA1*. Also upregulated were numerous modulators of FGF signalling, including *SPRY1, SPRY2*, *SPRED1, SPRED 2*, and *DUSP6*. Upregulated transcription factor genes included the T-Box genes *T*, *TBX4*, *TBX6 *and *EOMES*, the ETS factors *ETV1, ETV4 *and *ELK3*, several homeobox-containing genes such as *DLX1 *and *MKX*, as well as *SNAI2, ZIC3, ATF3, ATF4, XBP1*, and *POU3F1 *(*OCT6*).

**Table 1:** Summary of Changes in Regulatory Gene Expression

Gene Name	Reference Sequence	Preingression Vs. Lateral Epiblast	Preingression Epiblast + SU5402 (FGFR)	Preingression Epiblast + U0126 (MAPK)	Preingression Epiblast + LY294002 (PI3K)
**T **T, brachyury homolog	NM_204940	U^1,3^	D^1,2,3^	D^1,2,3^	NC^1,2,3^

**TBX4 **T-box 4	NM_001030537	NC^1^	D^1^	NC^1^	D^1^

**TBX6 **T-box 6	NM_001030367	U^1,2,3^	D^1, 2,3^	NC^1,2,3^	D^1,2,3^

**EOMES **eomesodermin homolog	XM_426003	U^1^	D^1,2^	D^1^	D^1^

**ETV1 **ets variant gene 1 (Er81)	NM_204917	U^1,2,3^	D^1,2^	D^1^	NC^1^

**ETV4 **ets variant gene 4 (Pea3)	XM_418106	NC^1,2,3^	D^1,2,3^	D^1,3^	NC^1,3^

**ELK3 **ETS-domain protein (SRF accessory protein 2)	NM_001030749	U^1,3^	D^1,2^	D^1^	NC^1^

**EVX1 **even-skipped homeobox 1	XM_425994.2	NC^1^	D^1,2^	D^1^	D^1^

**DLX1 **distal-less homeobox 1	NM_001045842.2	NC^1^	D^1^	NC^1^	D^1^

**TLX3 **T-cell leukemia homeobox 3	XM_001233188.1	U^1^	D^1^	ND	ND

**CDX4 **caudal type homeobox 4	NM_204614.1	U^3^	D^3^	D^3^	ND

**SNAI2 **snail homolog 2	XM_419196.2	U^2,3^	D^2,3^	D^3^	D^3^

**ZIC3 **Zic family member 3	AF188736	NC^1^	D^1^	D^1^	NC^1^

**JAZF1 **JAZF zinc finger 1	XM_418732.2	NC^1^	D^1^	NC^1^	D^1^

**XBP1 **X-box binding protein 1	NM_001006192.1	U^1^	D^1^	D^1^	D^1^

**ATF3 **activating transcription factor 3	XM_419429.2	U^1^	D^1^	D^1^	NC^1^

**ATF4 **activating transcription factor 4	NM_204880.1	U^1^	D^1^	D^1^	D^1^

**POU3F1 **POU domain, class 3, transcription factor 1 (Oct 6)	XM_427826.1	NC^1,3^	D^1^	D^1^	D^1^

**HDAC7A **histone deacetylase 7A	NM_001031402.1	NC^1^	D^1^	NC^1^	D^1^

**HDAC8 **histone deacetylase 8	XM_420178.2	NC^1^	D^1^	D^1^	D^1^

**FGF3 **fibroblast growth factor 3	NM_205327.1	U^1,^	D^1^	D^1^	NC^1^

**FGF4 **fibroblast growth factor 4	NM_001031546.1	U^1,2,3^	D^1,2,3^	NC^1,3^	D^3^

**FGF8 **fibroblast growth factor 8	NM_001012767.1	U^1,3^	NC^1,2,3^	NC/U^1,3^	NC^1,3^

**FGF18 **fibroblast growth factor 18	NM_204714.1	U^1^	D^1^	D^1^	NC^1^

**SPRY1 **sprouty homolog 1, antagonist of FGF signalling	NM_001097524	U^1,2,3^	D^1,2,3^	D^1,3^	NC^1,3^

**SPRY2 **sprouty homolog 2	NM_204800.1	U^1,3^	D^1,3^	D^1^	D^1^

**SPRY3 **sprouty homolog 3		U^1^	ND	D^1^	NC^1^

**SPRED2 **sprouty-related, EVH1 domain containing 2	XM_419341.2	U^1^	D^1^	D^1^	NC^1^

**DUSP6 **dual specificity phosphatase 6	NM_204354	U^1,3^	D^1^	D^1^	NC^1^

**IL17RD **interleukin 17 receptor D (SEF)	NM_204515.1	NC^1^	D^1^	D^1^	NC^1^

**WNT3 **wingless-type MMTV integration site family, member 3	NM_204675	U^1,3^	NC^1^	NC^1^	NC^1^

**WNT8A **wingless-type MMTV integration site family, member 8A	NM_205531.1	U^1,2,3^	D^1,2,3^	D^1,2,3^	D^1,2,3^

**WNT5B **wingless-type MMTV integration site family, member 5B	NM_001037269.1	U^1,2,3^	D^1,2,3^	D^1,3^	NC^1,3^

**FZD7 **frizzled homolog 7	NM_204221.1	U^1,3^	D^1^	NC^1^	NC^1^

**LOC417741 **similar to secreted Xwnt8 inhibitor sizzled	NM_204675	NC^1^	D^1^	D^1^	D^1^

**NOTCH1 **Notch homolog 1, translocation-associated	XM_415420	U^1,2,3^	D^1,2,3^	D^1,2,3^	NC^1,2,3^

**DLL1 **delta-like 1	NM_204973.1	U^1,2,3^	D^1,2,,3^	D^1,3^	NC^1,3^

**SNW1 **SNW domain containing 1	BX931222	U^1^	D^1^	NC^1^	D^1^

**NET1 **neuroepithelial cell transforming gene 1	NM_001030648.1	NC^1^	D^1^	D^1^	D^1^

**CER1 **cerberus 1, cysteine knot superfamily, homolog	NM_204823.1	U^1,3^	D^1^	U^1^	D^1^

**CFC1 **cripto, FRL-1, cryptic family 1	NM_204700.1	U^1^	D^1^	D^1^	NC^1^

**EPHA1 **EPH receptor A1	NM_204360.1	U^1,2,3^	D^1,2,3^	D^1,3^	D^1,3^

**EDNRB **endothelin receptor type B	XM_417001.2	U^1^	D^1,2^	D^1^	D^1^

**EDNRB2 **endothelin receptor B subtype 2	NM_204120.1	U^1,3^	D^1,3^	D^1^	NC^1^

**PDGFRA **platelet-derived growth factor receptor, alpha polypeptide	NM_204749.1	U^1,3^	D^1,3^	D^3^	NC^3^

**ROR1 **receptor tyrosine kinase-like orphan receptor 1	NM_204509.1	U^1^	D^1^	D^1^	D^1^

U: Gene Expression Increased. D: Gene Expressed Reduced. NC: No Change. ND: Not determined

Superscripts: Result Confirmed by: 1. Microarray; 2. PCR; 3. In Situ Hybridization

**Figure 6:**
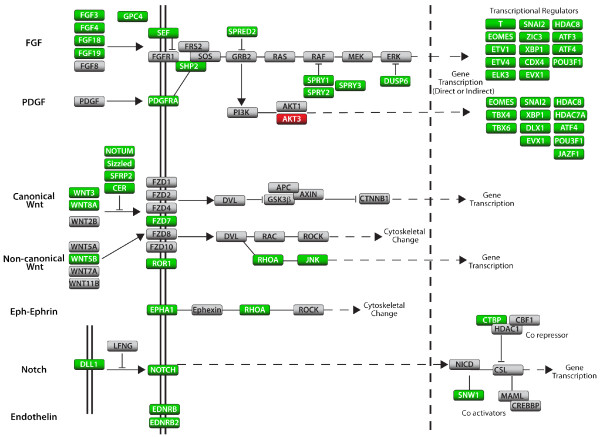
**Changes in mRNA levels among various signalling pathway members following inhibition of FGFR activity**. Representative aspects of each pathway are presented to illustrate FGFR dependent changes in gene expression. Genes shown in green require FGF signalling for expression. Genes shown in gray are expressed in the primitive streak but expression is not dependent on FGF signalling. Red are genes upregulated following FGF signalling inhibition.

A second series of microarray studies were performed to extend the ISH studies above in identifying changes in gene expression in preingression epiblast (excluding the primitive streak) of control embryos versus embryos treated with SU5402, U0126, or LY294002 (Table 1; Additional files 3, 4, 5, Tables S2-S4). Pairwise comparisons of mRNA levels in preingression epiblast from control versus treated embryos showed that expression levels of more than 500 genes were downregulated in the preingression epiblast following inhibition of FGFR kinase activity by SU5402 treatment (Additional file 3, Table S2). Using gene ontology terms to identify regulatory molecules, FGF signalling was found to regulate numerous ligands, receptors and pathway modulators of several signalling pathways (Table 1; Figure 6). Of the five FGF ligands expressed in the primitive streak and preingression epiblast, four (*FGF3*, *FGF4*, *FGF18 *and *FGF19*) were downregulated by SU5402 treatment while *FGF8 *was expressed at control levels. Expression of core components of the FGF signalling pathway was generally unaffected, while numerous positive and negative FGF signalling modulators were downregulated. Members of both the canonical and non-canonical WNT pathways were also downregulated, as were numerous negative regulators of WNT signalling (Figure 6). Expression of *RHOA *and *JNK *was also dependent on FGFR activity. Consistent with ISH results presented above, the *NOTCH1 *receptor and *DLL1 *ligand were highly downregulated, as were the NOTCH pathway transcriptional co repressor *CTBP *and the co activator *SNW1*. Additional signalling pathway genes downregulated by FGFR inhibition included *EDNRB, EDNRB2, PDGFRA*, and *EPHA1*. Further confirmation of these results was obtained by realtime RT-PCR analysis (Table 1; Additional file 6, Figure S2).

Comparison of expression changes in the preingression epiblast obtained with SU5402, U0126, and LY294002 revealed several patterns of gene regulation (Table 1; Additional files 3, 4, 5, Tables S2-S4). Most but not all of the genes downregulated by SU5402 were also downregulated by U0126. This included all identified FGF pathway members except for *FGF4 *which was unaffected by U0126, and *FGF8 *and *FGFR1 *which were unaffected by either inhibitor. Expression of the three ETS factors *ETV1, ETV4 *and *ELK3 *was also dependent on MAPK signalling. Expression of some transcription factors within the same family showed a differential response to the two inhibitors. For example, within the T-box transcription factor family, *T *and *EOMES *were highly downregulated by U0126 treatment, while *TBX4 *and *TBX6 *transcript levels were not affected. Similarly, the homeobox containing genes *DLX1 *and *MKX *were unaffected by U0126 despite being downregulated by SU5402. In fact, expression of *TBX4*, *TBX6, DLX1*, and *MKX *required PI3K signalling, while being independent of the MAPK pathway. A few genes required signalling through both pathways for expression (for example *EPHA1, EVX1*, *SPRY2*, *SZL*, and the preingression epiblast expression of *SNAI2*).

## Discussion

The role of FGF signalling in regulating gastrulation has been investigated in several classes of organisms. In frogs, FGF ligands can induce mesoderm in animal cap assays, and FGFR function and downstream pathway activity is required for mesoderm formation [39-41]. FGFs can induce mesoderm in chicken epiblast [25,26], and inhibition of FGF signalling blocks appearance of the primitive streak [6]. In mice, *FgfR1 *null embryos form a primitive streak, however primitive streak cells fail to express *Snai1*, to downregulate E-cadherin, or to undergo EMT [31].

In the present study, we find that blocking FGFR activity during gastrulation in chicken embryos also inhibits cell migration through the primitive streak. However, E-cadherin expression is not increased in SU5402 treated embryos, even in the preingression epiblast where *SNAI2 *expression is reduced. Although loss of E-cadherin is a primary requirement for EMT in numerous contexts, immunofluoresence analyses presented here and by others [23] show that EMT during avian gastrulation is not temporally linked with downregulation or altered intracellular localization of E-cadherin protein. EMT during gastrulation is closely associated with upregulation of N-cadherin in emerging mesoderm and endoderm cells, while E-cadherin protein levels decline only gradually as cells move to lateral regions of the embryo. E-cadherin levels also gradually decline in the presomitic mesoderm. Over expression of three different SNAIL proteins also failed to alter E-cadherin protein levels or localization in epiblast, primitive streak, or mesoderm cells. These findings do not rule out more subtle changes in E-cadherin function in the primitive streak unrelated to protein expression levels or localization detectable by confocal microscopy. The temporally controlled pharmacological approach used in this study may not be directly comparable to the FGFR gene ablation studies reported in mice [27]. Nevertheless, it appears that there are significant differences between chickens and mice in the regulatory pathways downstream of FGF signalling controlling the movement of cells through the primitive streak. Evidence presented here suggests that FGF-dependent pathways controlling EMT are independent of changes in E-cadherin expression, and furthermore that loss of E-cadherin is not temporally associated with EMT.

How, then, is FGF signalling regulating the movement of cells through the chicken embryo primitive streak? The broad requirement of FGF signalling for expression of components of numerous regulatory pathways during avian gastrulation suggests that FGF signalling may co-ordinately control multiple pathways related to the EMT process. RHOA regulation of microtubule dynamics is required to regulate basement membrane breakdown and EMT during avian gastrulation [23], and non-canonical WNT signalling is necessary for cells to transition from epiblast through the primitive streak to the mesoderm [16]. While direct regulation of these pathways by FGF signalling has not been addressed, expression of pathway components (*RHOA *and *NET1*; non-canonical *WNT5B*) is regulated by FGFR activity (Figure 6; Table 1; Additional file 3, Table S1; Additional file 6, Figure S2). Expression of the EPHA receptor, *EPHA1*, in preingression epiblast and primitive streak is also dependent on FGF signalling. Preliminary studies indicate that its function is also necessary for cells to undergo gastrulation (K.M. Hardy, P.B. Antin, unpublished observations). N-cadherin expression is also dependent on FGFR activity. Recent findings have shown that N-cadherin expression is required for cells to properly migrate away from the primitive streak [18]. The intracellular FGF signalling antagonists SPROUTYs and SPREDs contribute to the coordinate regulation of mesoderm induction and cell movement by differentially regulating signalling downstream of the FGF receptor-ligand interaction. SPROUTYs antagonize PLCγ signalling to regulate convergent extension, while SPREDs regulate the RAS/MAPK pathway to modulate gene expression [42]. In the chicken preingression epiblast, FGFR/RAS/MAPK signalling regulates expression of *SPROUTY1, SPROUTY2*, *SPROUTY3*, and *SPRED2*, suggesting that both pathways are activated. Although roles for NOTCH, ENDOTHELIN and canonical WNT pathways in regulating EMT during avian gastrulation have not yet been delineated, major components of each pathway are regulated by FGF signalling and each has been shown to regulate aspects of EMT in other contexts [43,44].

It is intriguing that all FGF ligands examined except *FGF8 *are downregulated following inhibition of FGF signalling. Mechanisms regulating its expression are not known. Several laboratories have shown that *FGF8 *is required for mesoderm cells to migrate away from the primitive streak, but not for EMT. Mouse embryos lacking *FGF8 *(which also fail to express *FGF4 *in the primitive streak) show normal EMT within the primitive streak, however mesodermal cells fail to migrate away from the midline [32]. It is possible that both ligands regulate the migration of mesoderm cells, because, in chicken, the lateral migration of mesoderm cells is directed towards a source of FGF4 but away from FGF8 [21]. How cells achieve this directional migration is unclear. Emerging mesoderm cells downregulate FGF receptor expression (Figure 1; [17,20]), although they become re-expressed as cells move to more lateral regions.

FGFR activity in the preingression epiblast controls both the RAS/MAPK and PI3K/AKT pathways. Some genes are regulated only through one pathway, while a few genes require both pathways for expression. For example, *T *expression is mediated through RAS/MAPK but is independent of PI3K/AKT signalling, while the related T-Box factors *TBX4 *and *TBX6 *require PI3K/AKT signalling but are independent of the RAS/MAPK pathway. The T-Box factor *EOMES *requires both pathways for expression. The regulation of *SNAI2 *expression by FGFR signalling is particularly interesting. Following inhibition of FGFR kinase activity (or inhibition of MEK or AKT activity), *SNAI2 *expression is downregulated in the preingression epiblast but not in the primitive streak or mesoderm. This finding supports the concept of modular regulation of gene expression in the primitive streak region (Figure 1), and also highlights species-specific differences in the regulation of *SNAIL *genes.

Collectively, the FGFR inhibitor SU5402, the MEK inhibitor U0126, and PI3K inhibitor LY294002 have been used in a large number of published studies to investigate FGF signalling pathways. However, in some contexts, each can inhibit other pathways, and multiple signalling pathways can signal through MEK and/or PI3K. SU5402, for example, can also inhibit the activity of VEGFR2. In this study, it is highly unlikely that VEGF signalling rather than FGF signalling is regulating the pathways shown to be affected by SU5402, because VEGFR2 is not expressed in the avian primitive streak and the VEGFR inhibitors SU1498 and SU5406 fail to reduce expression of *TBX6 *or *T *in the primitive streak (data not shown). Since MEK and AKT can act downstream of pathways other than FGF signalling, we have limited our comparisons of genes regulated by U0126 and LY294002 to those that are also regulated by SU5402.

Although in this study we have focused on genes that are positively regulated by FGF signalling, the preingression epiblast-specific downregulation of *SNAI2 *indicates that another likely function of FGF signalling is to repress gene expression in cells moving from the lateral to preingression epiblast. At least 600 genes are downregulated in the preingression epiblast versus lateral epiblast, and at least 300 of these genes are upregulated following SU5402 treatment. *SNAI2*, and perhaps other transcriptional repressors, might also function to repress the transcription of genes that would preclude transition from epiblast to mesoderm and endoderm. These transcription factors may also repress genes that are upregulated by gastrulation signals but whose precocious expression prior to entering the streak would be detrimental. While additional studies will be required to identify the underlying biological significance, FGF signalling in the preingression epiblast both activates and represses gene expression.

## Conclusions

We have shown that FGF signalling is required for the movement of cells from the epiblast through the primitive streak to the mesoderm of gastrula stage chicken embryos. FGF dependent mechanisms regulating migration are independent of apparent alterations in E-cadherin protein expression or localization. Further, the levels and intracellular localization of E-cadherin do not appear to change as cells undergo EMT during gastrulation. FGF signalling positively and negatively regulates the expression of a large number of genes in the preingression epiblast, primitive streak and newly formed mesoderm layer (Figure 6; Table 1). These include members of several major signalling pathways, among them the FGF, canonical and non-canonical WNT, NOTCH, PDGF, EPH-EPHRIN, and ENDOTHELIN pathways. A large number of transcriptional regulatory factors are also regulated by FGFR activity, and well as the cell adhesion molecule, *N-cadherin*. Of pathways known to regulate cell migration through the primitive streak, FGF signalling regulates the expression of components of several, including *RHOA *and non-canonical *WNT5B*.

## Methods

### Embryo culture and pharmacological treatments

Fertile chicken (*Gallus gallus*) eggs were obtained from Hy-Line International (Spencer, IA) incubated 37°C in a humid environment until Hamburger-Hamilton (HH) stage 3d-4 [45,46]. Embryos were removed from the egg and cultured in modified New culture on egg agar plates [47]. Embryos were submerged in 100 μM SU5402 (Pfizer, New York, NY), 100 μM U0126 (Promega, Madison, WI), 100 μM LY294002 (Invivogen, San Diego, CA) or DMSO carrier diluted in cell culture medium supplemented with penicillin, streptomycin and glutamate (Invitrogen). Incubation of embryos younger than stage 3d-4 in the inhibitors led to highly impaired development, and so only stage 3d-4 embryos were used for these studies. Embryos were incubated for 5 hours in a cell culture incubator at 37°C, and then were either fixed in 4% paraformaldehyde in PBS (PFA) and processed for ISH, or microdissected into an NP-40 extraction buffer and processed for western blot analysis. In GFP cell migration studies, stage 3d embryos were pretreated with SU5402 or DMSO for 2 hours prior to electroporation, and then were reincubated in SU5402 or DMSO for a further 5 hours before fixation.

### Whole-mount in situ hybridization and PCR

Embryos at the desired stage were either directly fixed in 4% PFA overnight at 4°C, or were subjected to treatment conditions and then fixed. Embryos were prepared for hybridization essentially according to Nieto et al. [48], but with minor modifications. Digoxigenin-labelled RNA probes were generated with the following linearizing restriction enzymes (Invitrogen, Carlsbad, CA) and RNA polymerases (Roche, Indianapolis, IN): *T *(R. Runyan, University of Arizona), HindIII/T3; *DLL1 *(BBSRC), Not1/T3; *EPHA1 *[13], EcoR1/T7; *EFNB2 *[13], EcoR1/T7; *FGF4*, *FGF8 *(G. Schoenwolf, University of Utah), EcoR1/T7; *FGFR1*, *FGFR2*, *FGFR3 *(K. Storey, University of Dundee), Xho1/T3; *NOTCH1 *(BBSRC), Not1/T3; *PDGFRA *(BBSRC), Not1/T3; *SNAI2 *(University of Delaware), Not1/T3; *TBX6*, Xba1/T7; *WNT5B *(S. Chapman, Clemson University), EcoR1/T3; *WNT8A *(K. Yutzey, Cincinnati Children's Medical Center), Sph1/SP6.

Embryo cell layers were isolated from control and treated embryos using electrolytically sharpened tungsten needles, then placed in TRIZOL reagent (Invitrogen) and total RNA isolated. RNA concentrations were determined using a Nanodrop, and RNA was stored in DEPC treated H_2_0 at -80°C for up to three months. cDNA was transcribed using the iScript cDNA synthesis kit (Bio-Rad, Richmond, CA). Intron spanning PCR primers were designed using MacVector software. Accession numbers of the mRNA sequences used for primer design, primer sequences, and PCR product lengths are provided in Additional file 7, Table S5. Realtime PCR assays were performed in triplicate, including no template controls, in a Rotorgene Q PCR machine using standard protocols and the Rotorgene statistical analysis software. PCR products were sequenced to confirm identity. Following assessment of several candidate reference genes, hydroxymethylbilane synthase (HMBS) was chosen because HMBS mRNA levels were unchanged between control and experimental samples.

### Antibodies and western blots

Rabbit anti-GFP (Invitrogen) and rabbit anti-FLAG (Cell Signalling, Danvers, MA) were used at 1:500 for immunofluorescence. Mouse anti-E-cadherin (Cat. No. 610181; BD Biosciences, San Jose, CA) and mouse anti-N-cadherin (Sigma-Aldrich, St. Louis, MO) were utilized at 1:500 and 1:250 respectively for immunofluorescence. The mouse monoclonal antibody against E-cadherin was generated using the C-terminal 148 amino acids of human E-cadherin as the immunogen. This antibody recognizes a single band of 120 kD on western blots of whole cell embryo lysates (data not shown), and has been used in other studies showing E-cadherin expression during chicken gastrulation [23]. Rabbit anti-pERK (phospho-p44/42), rabbit anti-ERK (p44/42), rabbit anti-pAKT, and rabbit anti-AKT (all Cell Signalling) were used at 1:1000 for western blotting. Goat anti-rabbit-AF488, goat anti-mouse-IgG_1_-AF594 and goat anti-mouse-IgG_2a_-AF488 (all Invitrogen) were used at 1:500 for immunofluorescence. Donkey anti-rabbit-HRP (Jackson ImmunoResearch, West Grove, PA) was utilized at 1:500 for immunohistochemistry and 1:7500 for western blotting.

For western blots, embryos treated with pharmacological inhibitors were washed with PBS, then endoderm and mesoderm was removed carefully removed with a sharpened tungsten needle. The primitive streak and preingression epiblast region from both sides of the primitive streak (epiblast directly adjacent to the primitive streak and extending the full length of the primitive streak excluding Hensen's node) was isolated from 12-14 control or treated embryos. Tissue was lysed in an NP-40 extraction buffer [49], and then proteins were separated on 7.5% SDS-PAGE gels and transferred to nitrocellulose membranes. Transfers were verified with PonceauS staining. Membranes were blocked and probed using standard protocols. Following protein detection, antibody conjugates were removed using Restore western blot stripping buffer (Thermo/Pierce, Rockford, IL), and verified by repeating secondary antibody and subsequent steps. Stripped membranes were then reprobed for either total ERK or AKT as a control.

### Electroporation, constructs, immunofluorescence, and cell analyses

Electroporation and subsequent immunofluorescence was carried out essentially as previously described utilizing the following conditions on an Intracel TSS20 Ovodyne electroporator: three 400ms pulses at 4V spaced 1s apart. Briefly, stage 3d embryos were electroporated by targeting the posterior epiblast. Under these conditions, only epiblast cells are electroporated [16]. pBE-WTcSNAI2 was created by cloning full-length chicken SNAI2 in place of GFP in the pBE vector. pBE-WThSNAI1 and pBE-6SAhSNAI1 were subcloned from the CMV-Tag2B vector (a gift of Dr. MC Hung, University of Texas MD Anderson Cancer Center, Houston, TX) into the pBE plasmid in place of GFP. All three of these sequences are flanked with a C-terminal FLAG tag for detection by immunofluorescence. Embryos in New culture were either: 1) electroporated with the pBE plasmid (GFP) or with pBE-WTcSNAI2, pBE-WThSNAI1 or pBE-6SAhSNAI1, and incubated in a cell culture incubator for 8 hours; or 2) pretreated for 2 hours with DMSO or SU5402, electroporated with the pBE plasmid and then reincubated for 5 hours. Following incubation, embryos were fixed and processed for immunofluorescence. Embryos were dehydrated through methanol and stored overnight at -20°C, then rehydrated and blocked in 5% goat serum in PBS-T for 1 hour at room temperature. Embryos were incubated in primary antibody diluted in block overnight at 4°C, then washed extensively and incubated in AlexaFluor-conjugated secondary antibody overnight at 4°C. Following extensive washing, embryos were imaged in whole mount on a Leica MZ16FA stereomicroscope, and then processed into Paraplast for sectioning at 8 μm. Transverse section images were captured on a Leica LeitzDMRXE compound microscope or on a Zeiss Meta510 confocal microscope.

Cell localization in the epiblast, primitive streak, and mesoderm was analyzed essentially as previously described [16]. Positive cells in these areas were counted for a region of ~100 μm from posterior expression, and results were presented as proportions of positive cells. EPIBLAST included preingression epiblast and lateral epiblast extending to the area pellucida-opaca border; STREAK included the primitive streak; MESODERM included the medial and lateral mesoderm regions to the area pellucida-opaca border (see Figure 1A for depiction of domains). Significant differences were calculated with the Student's T-test feature of Microsoft Excel. Standard deviations were calculated in Microsoft Excel.

### Microarray and pathway analyses

For gene expression comparison between lateral and preingression epiblast, lateral or preingression epiblast was microdissected from approximately 30 stage 4 embryos using electrolytically sharpened tungsten needles (see Figure 1A for depiction of domains). Cell layer fragments were placed in TRIZOL and RNA isolated according to standard protocols. RNA quantitation and integrity was determined using an Agilent Bioanalyzer. For gene expression comparison between control versus SU5402, U0126 or LY294002 treated embryos, embryo treatments were performed as described above. Five hours after treatment initiation, preingression epiblast was microdissected from 15-30 control or treated embryos and processed for RNA extraction. cRNA was extracted, amplified, labelled and hybridized according to standard protocols using dye swaps. All microarray studies were performed using a custom 20,477 feature 70-mer long oligo microarray printed in our laboratory. The probe set was developed by ARK-Genomics (http://www.ark-genomics.org/microarrays/bySpecies/chicken/) using chicken ENSEMBL transcripts, and covers much of the chicken genome. Normalization was performed according to a custom pipeline written in the R statistical computing language. Within chip normalization was performed using the R package OLIN [50]. Following normalization, false discovery rates were computed, and those spots demonstrating a location- or intensity-dependent bias (FDR > 1%) were subsequently masked from downstream analysis. Standard libraries in the R BioConductor package were then used to normalize between chips [50]. Finally, linear models were fit to the normalized gene expression data using the limma library, which computes log2 fold-change (logFC), indicating the direction and quantity of the differential gene expression between the samples, summary statistics including T- and B-statistics, and the adjusted p-value that takes into account the false discovery rate [51]. For each comparison in every study, Q-values were also computed using the R package qvalue. Pathways shown in Figure 6 are derived from the KEGG pathway database (http://www.genome.jp/kegg) and the published literature. Microarray results have been deposited in the NCBI Gene Expression Omnibus (GEO; accession #GSE27403)

## Authors' contributions

KMH participated in the experimental design, carried out most of the embryo experiments and drafted the manuscript. TAY participated in the experimental design and helped to carry out all of the embryo experiments. JHK helped to design the microarray studies and performed the statistical analyses of the microarray data. ASB performed the realtime PCR assays and data analysis. PBA conceived the study, participated in its design and coordination, conducted some of the embryo experiments and helped to draft the manuscript. All authors read and approved the final manuscript.

## Supplementary Material

Additional file 1 **Figure S1 E-cadherin and N-cadherin immunolocalization in control and SU5402 treated embryos**. Transverse sections through a control (A-G) and an SU5402 treated (A'-G') embryo, showing immunolocalization of E-cadherin (red) and N-cadherin (green) at different levels along the primitive streak. Section levels are shown on the corresponding whole embryo images. Click here for file

Additional file 2 **Table S1 List of genes whose expression levels increase or decrease between the lateral and preingression epiblast of stage 4 embryos**. Click here for file

Additional file 3 **Table S2 List of genes downregulated or upregulated in the preingression epiblast by SU5402 treatment**. Click here for file

Additional file 4 **Table S3 List of genes downregulated or upregulated in the preingression epiblast by U0126 treatment**. Click here for file

Additional file 5 **Table S4 List of genes downregulated or upregulated in the preingression epiblast by LY294002 treatment**. Click here for file

Additional file 6 **Figure S2 Realtime RT-PCR validation of Microarray and ISH expression analyses**. Realtime RT-PCR analyses of mRNAs levels in control versus SU5402 treated preingression epiblast. Data are presented as fold change in preingression epiblast mRNA levels of control versus SU5402 treated embryos. All samples were run in triplicate; standard deviations are shown. Ratios are compared to the control mRNA HMBS (hydroxymethylbilane synthase), the levels of which were not changed between control and SU5402 treated samples. Click here for file

Additional file 7 Table S5 Primer sequences used for realtime RT-PCR validation Click here for file
