# Supplementary figures and images for: FGF signalling through RAS/MAPK and PI3K pathways regulates cell movement and gene expression in the chicken primitive streak without affecting E-cadherin expression
Source: BMC Dev Biol. 2011 Mar 21;11:20. doi: 10.1186/1471-213X-11-20 (PMC3071786; doi:10.1186/1471-213X-11-20)

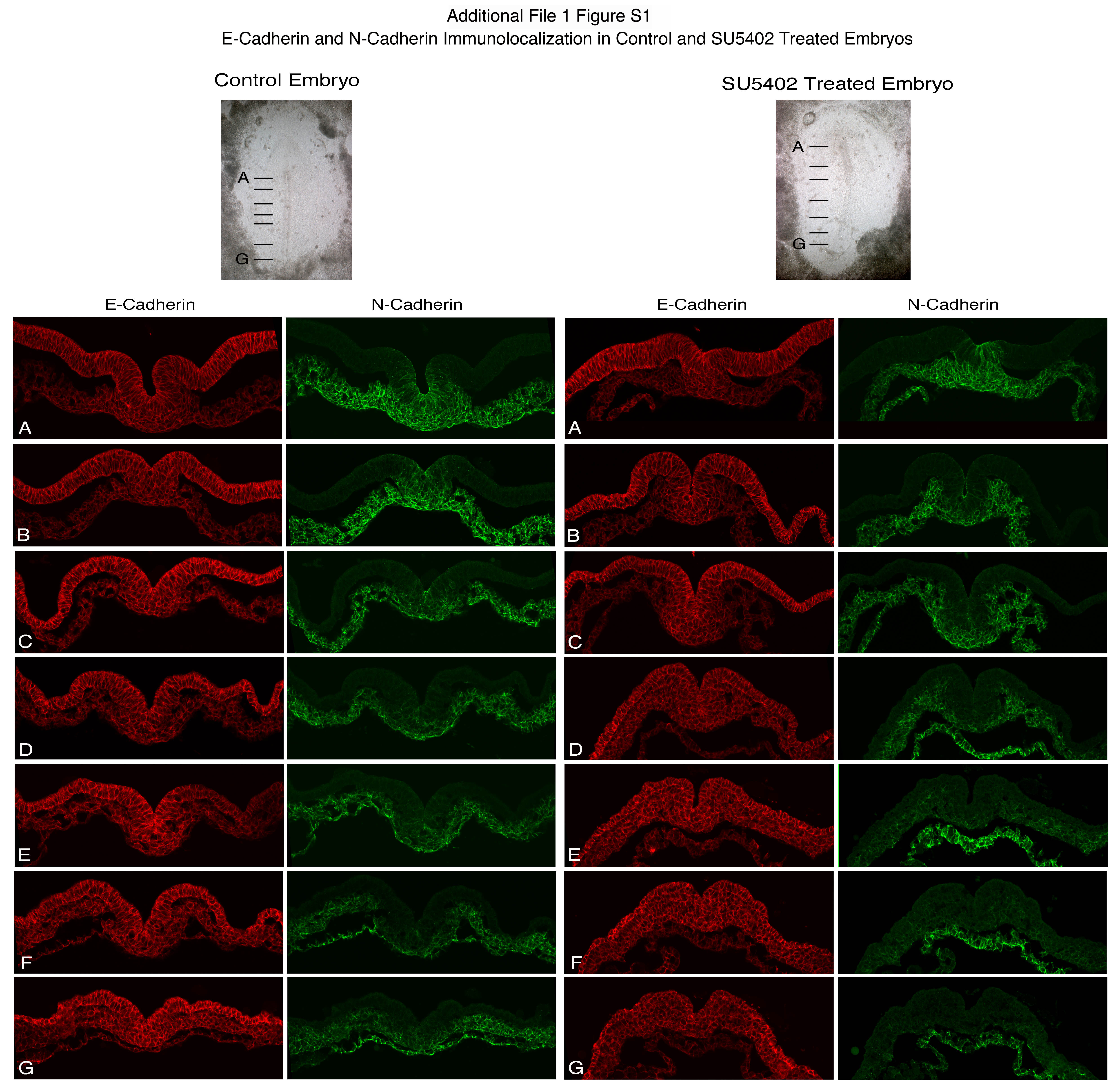

Supplement: Additional file 1 — Figure S1 E-cadherin and N-cadherin immunolocalization in control and SU5402 treated embryos. Transverse sections through a control (A-G) and an SU5402 treated (A'-G') embryo, showing immunolocalization of E-cadherin (red) and N-cadherin (green) at different levels along the primitive streak. Section levels are shown on the corresponding whole embryo images. [file 1471-213X-11-20-S1.JPEG]

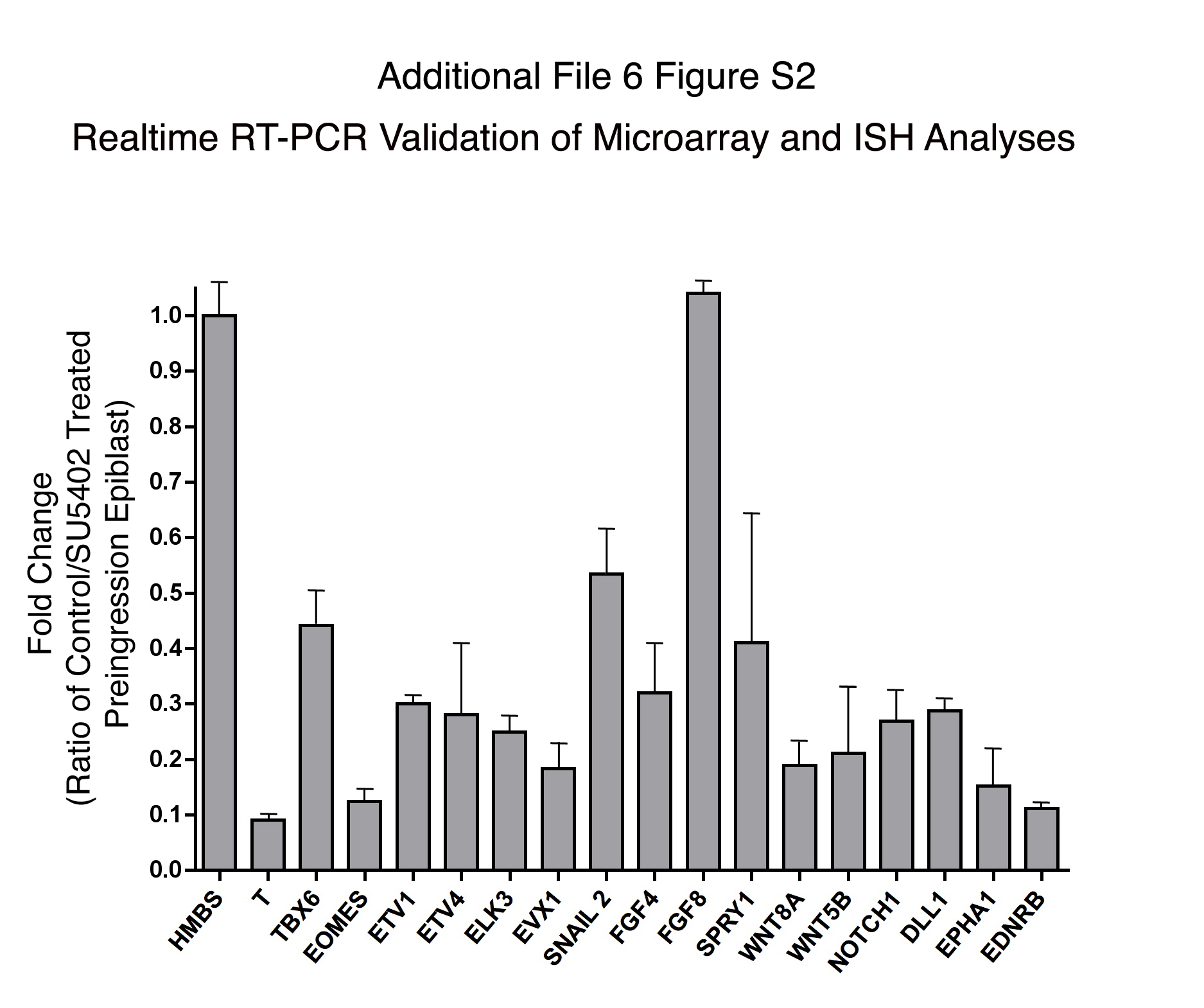

Supplement: Additional file 6 — Figure S2 Realtime RT-PCR validation of Microarray and ISH expression analyses. Realtime RT-PCR analyses of mRNAs levels in control versus SU5402 treated preingression epiblast. Data are presented as fold change in preingression epiblast mRNA levels of control versus SU5402 treated embryos. All samples were run in triplicate; standard deviations are shown. Ratios are compared to the control mRNA HMBS (hydroxymethylbilane synthase), the levels of which were not changed between control and SU5402 treated samples. [file 1471-213X-11-20-S6.PNG]
